# Redesigning Telemedicine for Traditional Chinese Medicine: Service Design Approach to Digital Transformation

**DOI:** 10.2196/76752

**Published:** 2025-10-28

**Authors:** Arisara Jiamsanguanwong, Romanee Luo, Ratchapoom Kaingam, Oran Kittithreerapronchai, Waratta Authayarat

**Affiliations:** 1 Department of Industrial Engineering, Faculty of Engineering, Chulalongkorn University, Bangkok, Thailand; 2 Human-Robot Collaboration and Systems Integration Research Unit, Chulalongkorn University, Bangkok, Thailand; 3 Department of Industrial Engineering, Faculty of Engineering, Burapha University, 169 Long-Had Bangsaen Road, Saensuk, Mueang, Chon Buri, 20131, Thailand, 66 38102222, 66 38393463

**Keywords:** digital transformation, efficiency improvement, service design, telemedicine, traditional Chinese medicine

## Abstract

**Background:**

With the rising global adoption of telemedicine, there is a crucial need to address inefficiencies and challenges in current service systems. This case study focused on enhancing the telemedicine service system of a traditional Chinese medicine clinic.

**Objective:**

The primary objective was to identify and address pain points and inefficiencies in the existing telemedicine system with the aim of streamlining service operations for the benefit of both patients and service providers.

**Methods:**

Through comprehensive service design analysis, including the creation of a customer journey map and a service blueprint, key areas for improvement were identified, and the service process was redesigned accordingly. A user-friendly web application was developed and evaluated using usability testing and satisfaction assessments. Participants took part voluntarily. Task testing was conducted using real-world scenarios, with index of item-objective congruence values ranging from 0.84 to 1.00. Participants were assigned role-specific tasks as either patients or service providers in a step-by-step format, followed by role-specific paper-based questionnaires.

**Results:**

The redesigned system successfully streamlined operations by automating processes and reducing task complexity, resulting in improved time efficiency for both user groups. Participants included 6 patients (aged 23‐54 years) and 7 service providers from various departments. Usability testing revealed a task success rate of 100% for all tasks among patients, coordinators, physicians, and finance personnel as well as 83.33% among pharmacists. Satisfaction outcomes were positive: patients reported a net promoter score of 67, whereas service providers reported a mean System Usability Scale score of 71.4 (SD 20.76).

**Conclusions:**

This study highlights the transformative potential of telemedicine in health care delivery. For patients, consolidating services into a single digital platform improved accessibility and ease of use. For service providers, the system reduced repetitive tasks and facilitated more efficient task completion. These findings demonstrate the effectiveness of service design methodologies in enhancing telemedicine systems, ultimately contributing to improved health care quality and patient outcomes.

## Introduction

Telemedicine, also known as telehealth or eHealth, was introduced in the 1970s as a means of delivering health care services over long distances using telecommunication and IT tools [1,2]. These tools include voice and video calls, SMS text messaging, remote monitoring, and virtual consultations [3]. The US Centers for Disease Control and Prevention has defined telemedicine as “healing at a distance” [4]. Today, telemedicine is used for a wide range of services, including diagnosis of common illnesses, follow-up consultations, psychotherapy, and chronic disease management [5].

The benefits of telemedicine are widely recognized. These include cost savings [6], improved access for patients with mobility limitations [7], and enhanced continuity of care while minimizing exposure during the COVID-19 pandemic [8,9]. The global health crisis in 2020 accelerated the adoption of telemedicine as health care systems sought to reduce viral transmission and maintain service continuity [4,10]. In the United States, telehealth visits increased by 50% in the first quarter of 2020 compared to the same period in 2019 and surged by 154% by week 13 of 2020 [11]. Despite this rapid uptake, much of the telemedicine infrastructure at the time was still in its early stages of implementation, hindered by limited understanding of the virus and system unpreparedness [12].

While the urgency of the pandemic necessitated swift development and deployment of telemedicine platforms, numerous challenges persist. These include concerns about patient-provider relationships, digital literacy, technical reliability, privacy and data confidentiality, reimbursement policies, physical examination limitations, and acceptability among special populations [3,13,14].

This study focused on the development and improvement of a telemedicine system within a traditional Chinese medicine (TCM) clinic in Bangkok, Thailand. The clinic serves approximately 5000 patients monthly and presents unique service characteristics distinct from conventional modern medicine. TCM diagnostics rely on observation of the patient’s physical appearance, vocal quality, pulse palpation, and tongue examination, whereas treatments often involve custom herbal preparations. These distinctive diagnostic and therapeutic practices introduce additional complexity in adapting telemedicine systems for TCM settings.

The clinic initially launched telemedicine services via telephone in April 2020, expanding to video consultations in March 2022. According to internal records, the number of telemedicine visits more than doubled from 1955 in 2020 to 3972 in 2021, with the age group from 41 to 50 years representing the largest proportion of users. Despite the growth, both patients and service providers reported difficulties. Patients experienced confusion regarding appointment scheduling and status updates, whereas staff encountered challenges related to fragmented communication channels, repetitive manual tasks, and workflow inefficiencies.

Given the known advantages of telemedicine [7] and the increasing demand for remote TCM services, this study aimed to systematically identify pain points in the existing telemedicine service system and redesign it to improve efficiency and user experience for both patients and service providers.

## Methods

### Investigation of Current Service Operation

#### Overview

The telemedicine service was investigated from both patient and service provider perspectives. Two hypothetical workflows were developed to visualize the end-to-end service interaction: a *customer journey map* representing the patient experience [15,16] and a *service blueprint* illustrating the provider-side operations [16,17]. These workflows were constructed using data collected from direct observations of the service operations, content review of the clinic’s website, and semistructured interviews with service providers.

The purpose of these visual tools was to map the sequence of interactions between patients and the service system, as well as among service providers, to identify pain points and service gaps [18]. The preliminary workflows were subsequently validated through in-depth interviews: 4 current telemedicine patients validated the customer journey map, and representatives from each relevant department (eg, registration, consultation, and pharmacy) reviewed and confirmed the accuracy of the service blueprint.

#### Barriers From the Patient Perspective

**Figure 1.**
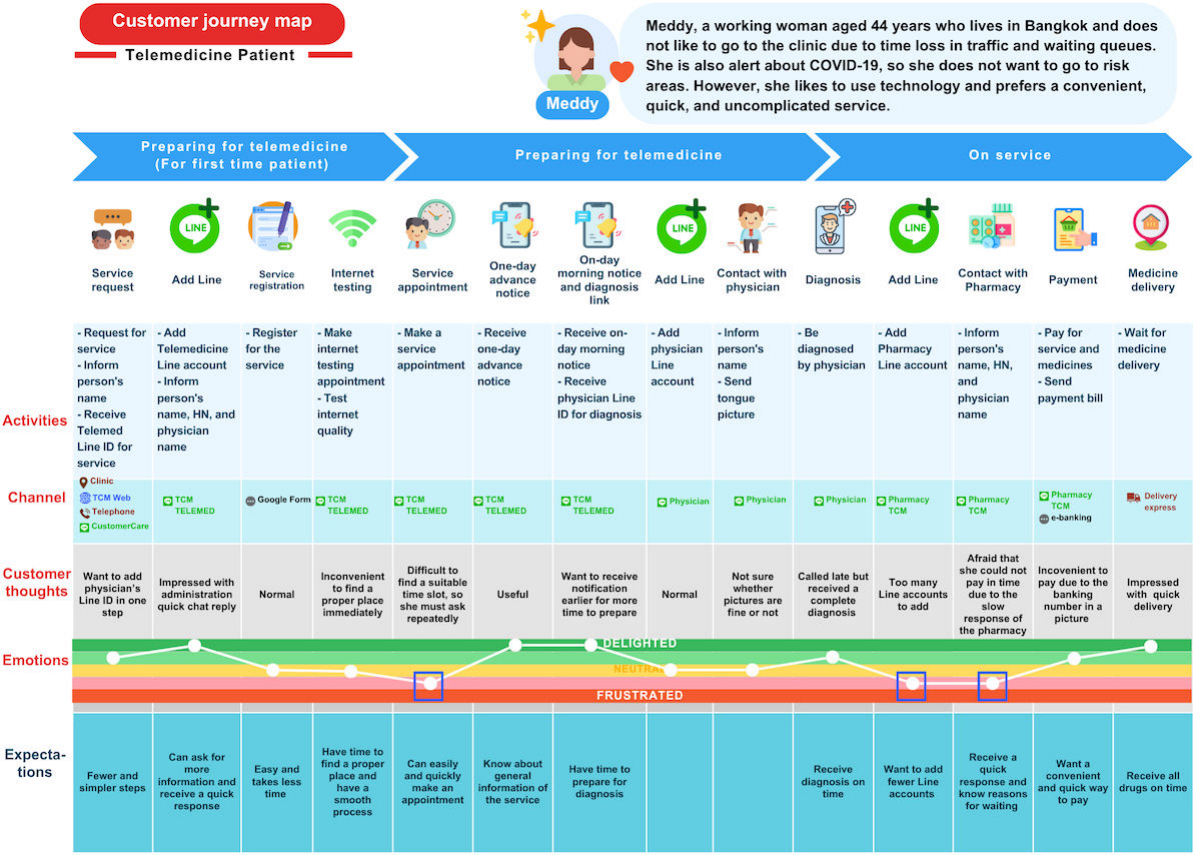
Customer journey map of the current telemedicine service (patient perspective). HN: health number; TCM: traditional Chinese medicine.

Figure 1 presents the validated customer journey map from the patients’ point of view. The analysis revealed 3 key barriers that contributed to user dissatisfaction, indicated by negative emotional states on the emotion-tracking row of the map.

First, patients experienced difficulty in scheduling appointments. As shown in the “Customer thoughts” row in Figure 1, many users had to engage in multiple rounds of communication with the clinic to secure a suitable time slot, some reporting up to 5 separate calls. This resulted in excessive human effort and a high interaction cost. Patients expressed a clear expectation for a simplified and more efficient appointment scheduling process.

Second, the current system required patients to interact with multiple communication channels. Specifically, they had to register with 4 separate Line messaging accounts (CustomerCare TCM, TCM TELEMED, the physician’s personal account, and Pharmacy TCM) to complete a single telemedicine session. This fragmentation added significant complexity and increased the interaction burden on patients. Many expressed the desire to minimize the number of communication accounts required for service access.

Third, there was a lack of transparency regarding service response times. Patients were not informed of estimated wait times for replies from service providers. Data logs revealed a wide variation in response time, ranging from 5 minutes to as long as 3 hours, with an average of 30 minutes. This uncertainty undermined patient confidence and satisfaction. Participants emphasized the need for real-time service tracking and automated notifications to improve the overall user experience.

Collectively, these issues of excessive communication effort, fragmented service channels, and uncertain wait times indicate critical inefficiencies in the current telemedicine system. From the patient perspective, three areas for improvement were identified: (1) simplification of the service process, (2) consolidation of communication channels, and (3) visibility and predictability of service status and wait times. Addressing these pain points through streamlined service design is expected to enhance patient satisfaction, reduce interaction costs, and improve the overall usability and efficiency of the telemedicine service.

#### Barriers From the Service Provider Perspective

**Figure 2.**
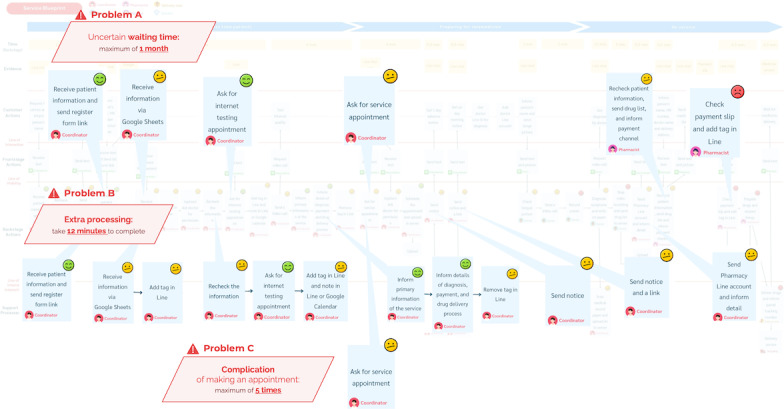
Service blueprint of the current traditional Chinese medicine telemedicine service (provider perspectives).

Figure 2 presents the current service blueprint for the TCM telemedicine operation, highlighting 3 primary barriers reported by service providers.

First, providers faced challenges due to uncertain waiting times. They were required to monitor patient responses closely and ensure timely replies to maintain patient satisfaction (problem A in Figure 2). Patient response times varied widely, ranging from as little as 10 minutes to as long as 1 month, with an average of 2 days. This unpredictability increased provider workload and disrupted workflow. Digitizing the system to automate follow-up communication would mitigate this issue.

Second, service providers were burdened by numerous repetitive administrative tasks (problem B in Figure 2). These included sending registration forms, checking for completeness, updating status tags, issuing notifications, and sharing Line contact details. Collectively, these tasks consumed approximately 12 minutes per patient, representing wasted time and avoidable effort. Automating these processes could substantially reduce inefficiency.

Third, appointment scheduling was unnecessarily complicated (problem C in Figure 2). Providers often engaged in repeated communication with patients (up to 5 exchanges to finalize a booking due to mismatched availability). This process increased provider effort and interaction costs, diverting attention from more value-adding clinical activities.

Overall, these barriers illustrate inefficiencies that compromise the effectiveness and stability of the telemedicine service. From the provider perspective, three key areas required improvement: (1) reducing waiting times, (2) eliminating repetitive administrative tasks, and (3) simplifying the appointment scheduling process.

**Figure 3.**
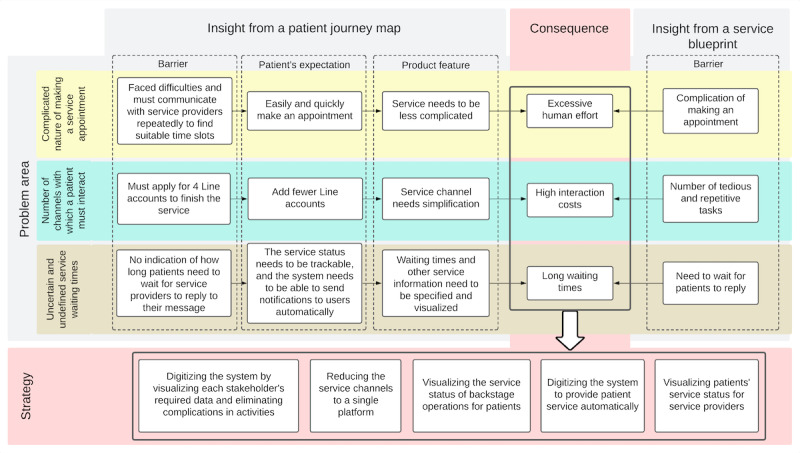
Common problems and improvement strategies for patients and providers.

When considered alongside patient-reported barriers, a common set of challenges emerged for both groups: (1) excessive human effort, (2) high interaction costs, and (3) long or uncertain waiting times. As shown in Figure 3, addressing these problem areas is essential to improving operational efficiency and ensuring a better overall telemedicine experience for both patients and service providers.

### System Design

#### Redesigning Service Operations

Following the identification of barriers in the current service system, solutions were designed to eliminate inefficiencies and enhance user experience. A digital transformation strategy was adopted integrating information, computing, communication, and connectivity technologies [19,20]. This approach aimed to strengthen the telemedicine service’s capabilities and address issues such as repetitive tasks and the difficulty of sharing real-time information between patients and providers. For example, dissatisfaction arising from uncertain waiting times could be mitigated by providing estimated wait times and visualizing them for users [21].

The success of such digital interventions also depended on staff skills and competencies. Therefore, comprehensive training for all system users was considered essential to ensure effective adoption of the redesigned processes.

**Figure 4.**
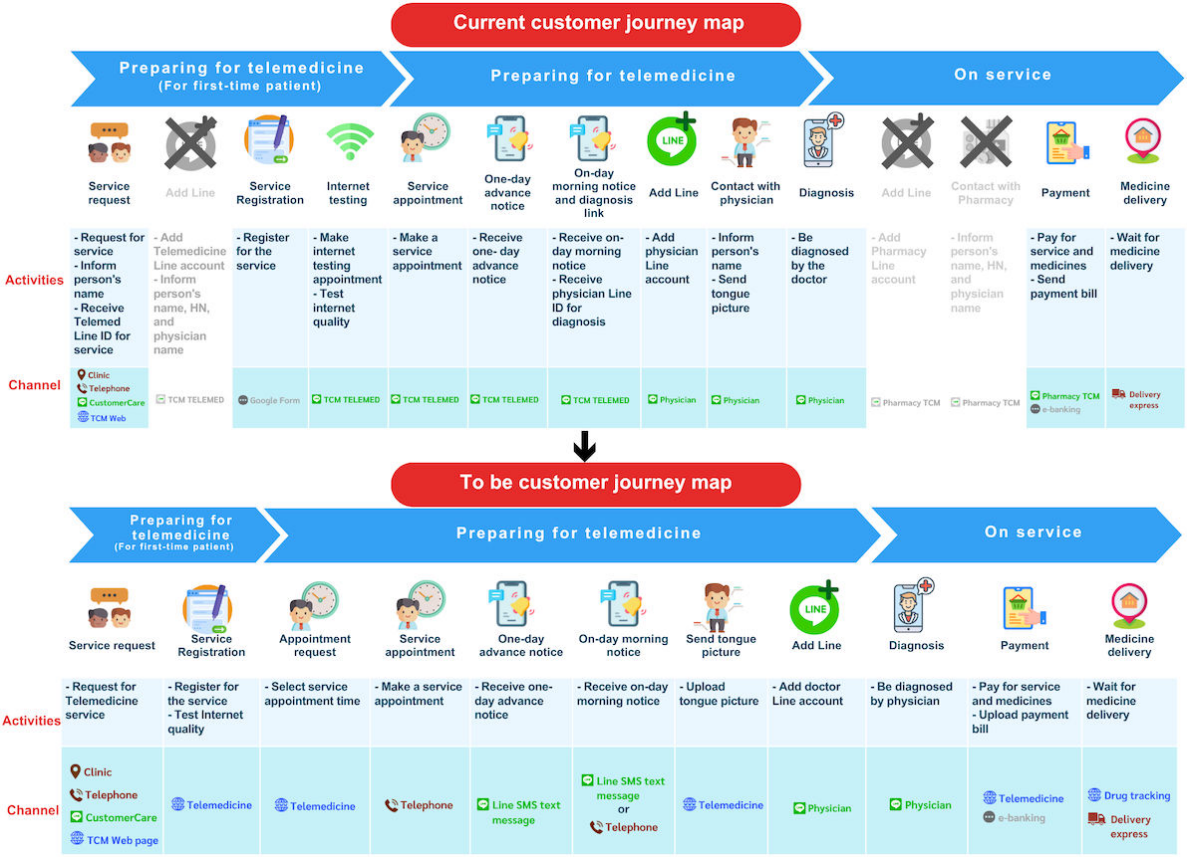
“To be” customer journey map of the redesigned telemedicine service (patient perspective). TCM: traditional Chinese medicine.

To guide implementation, a “to be” customer journey map was developed (Figure 4) illustrating three major strategies: (1) *channel consolidation* (consolidating multiple service channels into a single integrated platform that supported all service activities, thereby lowering patient interaction costs), (2) *system digitization* (streamlining complex activities such as appointment scheduling by digitizing workflows and visualizing the required data for each stakeholder), and (3) *service status visualization* (providing patients with real-time updates on backstage operations to reduce uncertainty and dissatisfaction caused by undefined waiting times [22]).

In summary, the redesigned service model focused on consolidating communication channels, digitizing processes, and visualizing service status—strategies aimed at streamlining operations, improving efficiency, and delivering a more seamless telemedicine experience.

**Figure 5.**
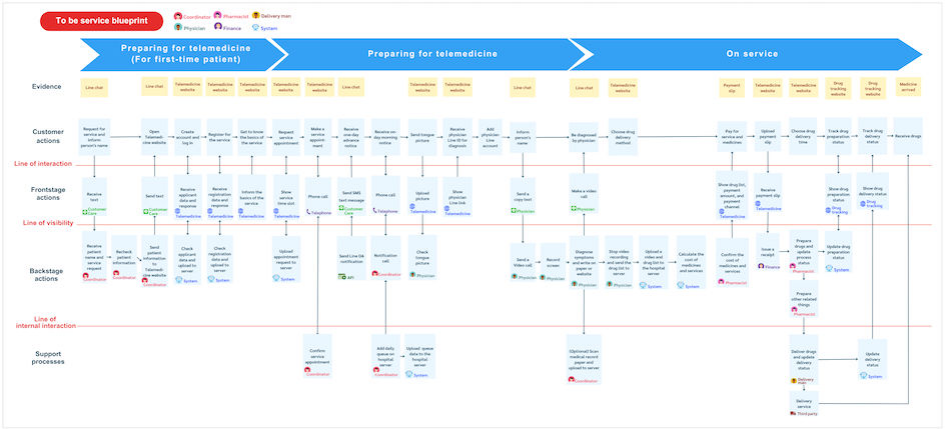
“To be” service blueprint of the redesigned telemedicine service (provider perspective). API: application programming interface; OA: official account.

Moreover, a “to be” service blueprint was developed to enhance the service provider experience (Figure 5, or see the enlarged image in Multimedia Appendix 1). This blueprint emphasized 2 key strategies. First, the system was digitalized to automate patient-facing processes, thereby reducing provider workload associated with repetitive administrative tasks and ongoing patient follow-up. Automation also eliminated complex tasks such as appointment scheduling. Second, the blueprint introduced service status visualization for providers accompanied by automated notification alerts when action was required. This functionality helped reduce uncertainty during patient response delays and minimized the need for providers to manually review patient messages. In summary, streamlining provider operations was achieved through 2 strategies: system digitalization and visualization of service status.

#### System Interface

The system interface is a critical component of service improvement, functioning as the primary channel of interaction between patients and health care providers. To guide interface design, a use case diagram of the redesigned telemedicine web application was developed to define user roles and their corresponding system functions. For example, users with system authentication privileges could manage log-in and log-out processes, change account passwords, create new patient accounts, and edit other user accounts.

Interfaces for each role were prototyped using Figma, a digital design platform that enables realistic mock-ups and supports iterative development [23]. Interactive buttons and workflows were tested during the prototyping stage, and feedback from trial users was collected to refine the design through iterative improvement cycles.

**Figure 6.**
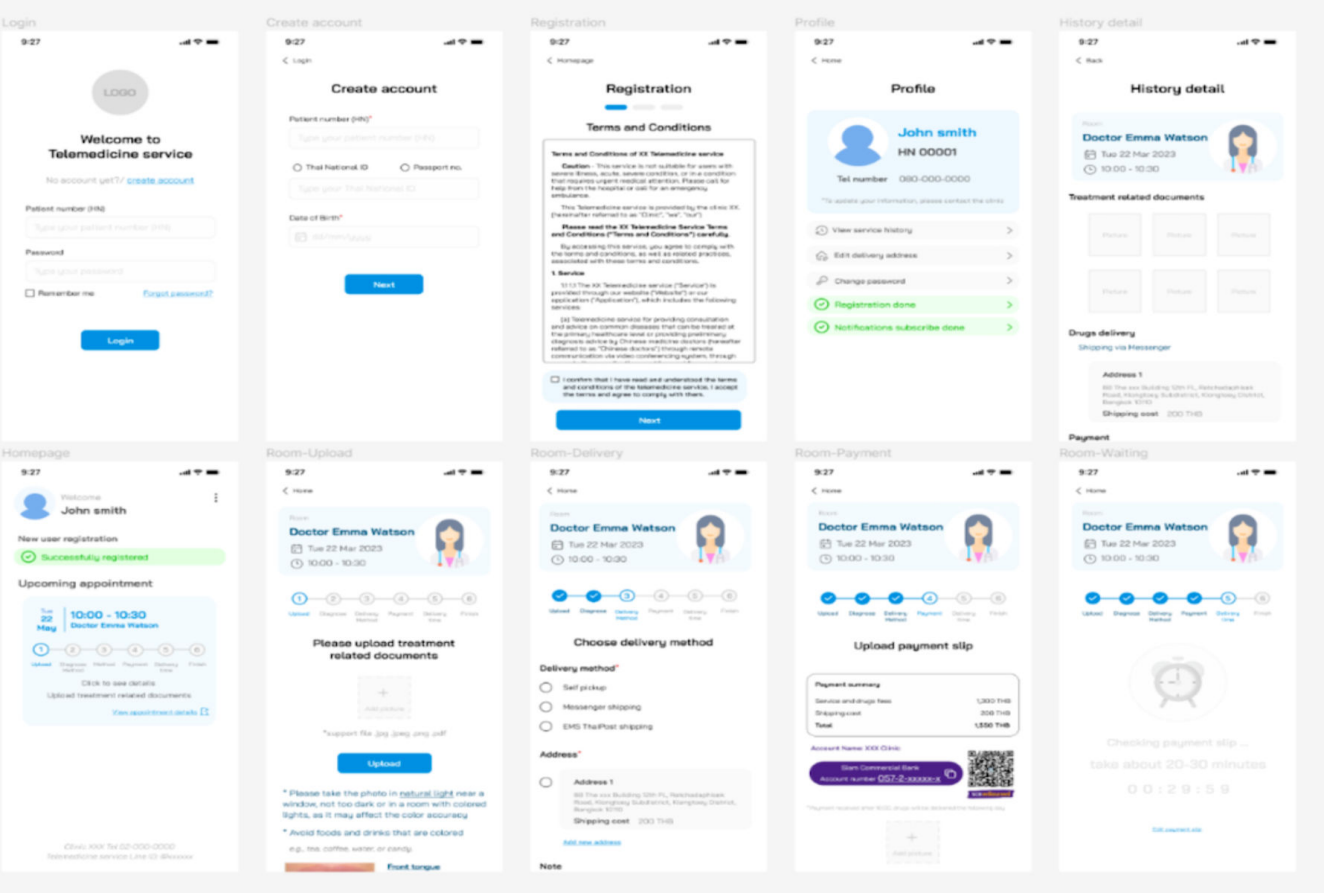
The patient web application interface optimized for mobile devices.

The final interface was divided into 2 dedicated websites: one for patients and one for service providers. The patient website was optimized for mobile devices and designed to support all essential patient tasks, including log-in, registration, profile management, and appointment booking (Figure 6, or see the enlarged image in Multimedia Appendix 2). In contrast, the provider website was optimized for desktop use to reflect providers’ routine work patterns. This platform supported role-specific functions for physicians, coordinators, pharmacists, and finance staff, facilitating streamlined management of patient services and system operations.

#### System Implementation

**Figure 7.**
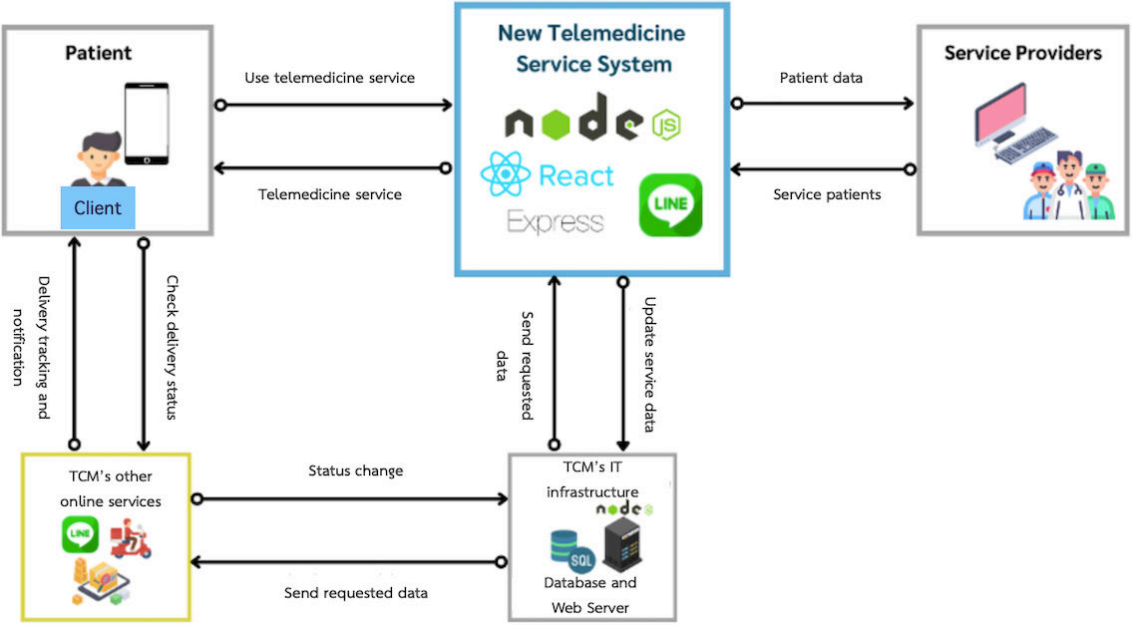
Overview of the redesigned telemedicine service system. TCM: traditional Chinese medicine.

Following the design phase, the next step was the implementation of the new telemedicine web application system. This stage involved translating the design concepts into a functional system accessible to both patients and service providers. An overview of the telemedicine service system is shown in Figure 7.

To support implementation, an entity-relationship diagram was developed to represent the database structure and the relationships between its entities [24]. The entity-relationship diagram served as a blueprint for database design, ensuring that data were logically organized and efficiently structured to support the system’s core functionalities.

In developing the TCM clinic’s telemedicine service system, the web applications described in the System Interface section were implemented using Node.js (version 10; OpenJS Foundation), a widely adopted runtime environment for web development. The system interfaces were designed to be dynamic and responsive, ensuring low latency and a positive user experience.

The implementation integrated several JavaScript libraries and packages. The front end was built using React (version 17), compatible with Node.js version 10, alongside additional libraries such as SweetAlert and Fortawesome to enhance interactivity and usability. For the back end, Express.js (OpenJS Foundation) served as the primary framework for building application programming interfaces that adhered to the Representational State Transfer architectural style, enabling communication between users and the clinic’s Microsoft SQL Server 2000 database. Supporting packages included *mssql* for database connectivity, *cors* to enable cross-origin requests, *express-fileupload* for image uploading, *bcrypt* for secure password encryption, and *line/bot-sdk* for integrating notification services through the Line app.

System interactions followed a client-server architecture: users engaged with the front-end interface, which transmitted data requests to the back-end system. The back end processed the data, executed the required operations, and returned results to the front end for user display.

Although the real-time communication module was developed independently from the back end, seamless integration allowed for direct connection between the web application and physicians’ Line accounts. Furthermore, the new telemedicine service system was linked with the clinic’s existing automated alert system, prescription tracking system [25], and medicine delivery service [26], ensuring compatibility with established workflows and continuity of care.

### System Validation

A system validation process was conducted to ensure that the redesigned telemedicine platform met the requirements of both patients and service providers and to evaluate user satisfaction with its performance and functionality.

#### Participants

Two participant groups were recruited: patients and service providers. Recruitment flyers were posted on noticeboards throughout the hospital where the TCM clinic is located. The flyers included details of the study objectives, experimental procedures, and eligibility criteria for both groups. Interested individuals self-screened for eligibility and contacted the research team via telephone to register.

A purposive sampling strategy was used to allow for in-depth understanding of user experiences [27]. Initial eligibility interviews were conducted by 2 web application developers from the author team. Participants were intentionally selected based on criteria relevant to the research objectives. To avoid bias, the remaining author team members who conducted the data analysis were blinded to the recruitment process.

#### Tasks

##### Overview

Task testing was conducted using simulated real-world scenarios designed to cover all key functions that patients and service providers would perform within the system. Separate test scenarios were developed for each role to ensure that all system features were evaluated comprehensively.

Content validity of the test scenarios was assessed by 3 domain experts, with each task receiving an index of item-objective congruence score between 0.84 and 1.00, indicating strong validity and suitability for use [28].

##### Tasks for the Patient Role

The patient scenarios were designed to address challenges commonly faced by individuals visiting the TCM clinic in person, particularly those who lived at a distance and found travel burdensome. Six scenarios were developed to validate the telemedicine functions from the patient perspective:

1. Registration: creating a telemedicine account and completing a 2-step registration process, including agreement to the telemedicine service terms and opting in for service notifications
2. Appointment booking: requesting a telemedicine consultation by selecting a specific date and time and choosing a physician from the provider list
3. Telemedicine consultation: completing the end-to-end telemedicine process, including uploading treatment-related documents (eg, tongue photos), joining the scheduled consultation, selecting a medicine delivery method, making payment, and choosing a delivery time
4. Address entry: adding a medicine delivery address with complete details (street address, district, province, and postcode)
5. Service history: viewing consultation history, including appointment details, physician information, diagnosis, payment records, and medicine delivery status
6. Password reset: resetting the telemedicine account password if forgotten

Collectively, these scenarios ensured validation of all core patient-facing functions in the telemedicine system.

##### Tasks for the Coordinator Role

The coordinator role scenarios reflected coordinators’ primary responsibilities, including reviewing service requests, managing appointments, and monitoring daily schedules. Four scenarios were developed to evaluate system functionality from the coordinators’ perspective:

1. Appointment creation: reviewing patient appointment requests, confirming the appointment with the patient via phone, and creating the appointment record in the system
2. Daily schedule review: viewing all patient service appointments scheduled for the day, including health number, name, phone number, date, time, and attending physician
3. Service status review: entering a patient’s health number in the search bar to access the profile page and view the current service status
4. Provider information management: editing a physician’s information in the system, such as Line ID and Line URL

##### Tasks for the Physician Role

The physician role scenarios represented physicians’ key responsibilities in reviewing patient appointments and treatment-related materials. Two scenarios were simulated:

1. Document reupload request: reviewing patient appointment details and treatment-related documents submitted via the system and then requesting patients to reupload documents when necessary
2. Service history review: accessing a patient’s historical records by entering the health number in the search bar to view profile information and past service data.

##### Tasks for the Pharmacist Role

Three scenarios were developed to evaluate the pharmacist role, which primarily involved managing patient medication and related communications:

1. Expense notification: viewing the patient’s health number, name, and delivery method and then entering service, medication, and shipping costs to inform the patient
2. Delivery detail review: accessing patient records to confirm delivery method and scheduled delivery time, including health number, name, phone number, and delivery type and time
3. Service status review: entering the patient’s health number in the search bar to access the profile page and review the current service status

##### Tasks for the Finance Personnel Role

Two scenarios were created to reflect the finance personnel’s responsibilities regarding payment verification and follow-up:

1. Payment reupload request: reviewing the “waiting for payment confirmation” list, assessing uploaded payment bills, and notifying patients when resubmission was required
2. Review of patients with unpaid consultations: viewing details of patients with unpaid consultations, including health number, name, phone number, and service date and time

### Measurement

Three quantitative usability metrics were used to evaluate task performance: success rate, number of interactions, and time on task. The success rate measured the effectiveness of the system and represented the ultimate outcome of usability testing. The number of interactions was used to evaluate efficiency by capturing the steps required for task completion. Time on task measured the time needed to complete each task and served as a primary indicator of efficiency [29]. Together, these metrics provided a comprehensive assessment of task effectiveness and overall system usability.

User satisfaction was assessed through 2 role-specific paper-based questionnaires. The patient questionnaire consisted of 4 sections: understanding of service procedures, ease of service completion, overall satisfaction, and net promoter score (NPS). The NPS, first introduced in 2003, measures customer loyalty by asking participants how likely they are to recommend a service to others, with scores ranging from –100 to +100 [30]. All patient questionnaire items were rated on a scale from 0 to 10, with higher scores indicating greater satisfaction.

The service provider questionnaire consisted of 10 items adapted from the System Usability Scale (SUS) [31]. The SUS includes statements about system usability rated on a 5-point Likert scale from “strongly disagree” (1) to “strongly agree” (5). Scores were converted to a percentile scale ranging from 0 to 100, with higher scores indicating better usability [31].

### Procedure

Testing was conducted using the provided mobile phone (participants could select either iOS or Android). Participants were given a scenario and a step-by-step list of tasks to complete, whereas their interactions with the interface were recorded. The focus of this study was to evaluate the usability of the telemedicine application, not the participants’ skills or knowledge.

Upon completing all scenarios and tasks, participants filled out a role-specific satisfaction questionnaire reflecting their experience with the telemedicine system. Finally, participants were thanked for their involvement and dismissed.

### Ethical Considerations

Ethics approval for this study was obtained from the Burapha University Ethics Committee for Human Research (approval HS 100/2567). All participants were comprehensively briefed in person regarding the procedural details and the data protection protocols implemented to ensure anonymity and subsequently provided informed consent before testing. The consent form stated that no personally identifiable information would be collected, testing would take approximately 30 minutes, and participants retained the right to withdraw at any time. It was explicitly communicated to participants that they retained the right to withdraw from the experiments at any moment without adverse consequences. Furthermore, participation was voluntary, with no compensation offered.

## Results

### System Design

One of the major advantages of the redesigned telemedicine system was its enhanced operational efficiency, achieved by applying insights from the customer journey map and service blueprint during the redesign process. By automating processes and reducing the number of required steps, the new system streamlined service operations and optimized task completion time for both patients and service providers.

From the patient perspective, the number of steps required to register for telemedicine services decreased from 3 to 1 (a 67% reduction). Similarly, the number of interactions required to book an appointment, make a payment, and enter delivery details decreased from 9 to 5 (a 44% reduction).

From the service provider perspective, efficiency gains were even more substantial. The number of steps required to register a patient, order testing online, and provide service details was reduced from 14 to 2 steps (an 86% reduction), saving approximately 10 minutes per patient. In addition, the number of steps required to schedule an appointment and prepare a patient for diagnosis decreased from 5 to 4 (a 20% reduction). The system also eliminated redundant tasks, such as adding patient tag statuses and sharing Line contact details of physicians, pharmacists, and finance personnel.

Collectively, these reductions enabled service providers to process requests more quickly and allocate more time to higher-value clinical tasks. Table 1 presents a comparison of interaction steps before and after system redesign from both the patient and provider perspectives.

**Table 1.** Improvement in interaction steps between users and the telemedicine system after redesign.

Tasks	Improvement in interaction steps		Reduction (%)
	Before redesign	After redesign	
Patient perspective			
Registering for telemedicine service	3	1	67
Communicating with the service provider during the pre- and postservice stages	9	5	44
Service provider perspective			
Registering, internet testing, and informing the patient of service details	14	2	86
Scheduling an appointment and preparing a patient for diagnosis	5	4	20
Adding the tag status of a patient in the system and sending the Line contacts of all stakeholders	5	0	100

### System Validation

A comprehensive validation process was conducted to evaluate the usability of the redesigned telemedicine service system. Usability was defined as “the extent to which a product or service can be used by specified users to achieve specified goals with effectiveness, efficiency, and satisfaction in a specified context of use” [32].

**Figure 8.**
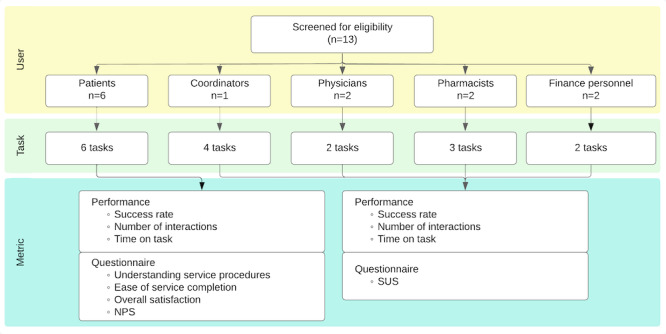
Number of participants in each role and details on their engagement. NPS: net promoter score; SUS: System Usability Scale.

Three categories of quantitative usability metrics were assessed: effectiveness (success rate), efficiency (time on task), and satisfaction (overall satisfaction rating, NPS, and SUS score). As suggested by Eysenbach [33], validation encompassed both task performance and subjective evaluation. Figure 8 summarizes the number of participants in each role, along with details of their engagement, including assigned scenarios, completion of paper-based questionnaires, and the measurement metrics.

A total of 13 participants were involved in this study. The patient group comprised 6 individuals (n=5, 83% female and n=1, 17% male), with an age range from 23 to 54 years (mean 36.5, SD 9.95 years). The service provider group consisted of 7 individuals representing various departments involved in the telemedicine service system: 1 (14%) female coordinator, 2 (29%) physicians (n=1, 50% male and n=1, 50% female), 2 (29%) female pharmacists, and 2 (29%) female finance personnel.

During the usability testing phase, all participants completed role-specific tasks using the redesigned telemedicine web application. The tasks were designed to cover all functional aspects of the system. Six participants evaluated the patient-facing web application, whereas 7 participants evaluated the provider-facing management platform. Descriptive statistics from the usability testing are shown in Table 2 (patient usability) and Table 3 (service provider usability).

**Table 2.** Patient statistical usability testing results (N=6).

Scenarios	Number of interactions		Time on task (s)		Task completion						Success rate (%)
	Mean (SD)	Range	Mean (SD)	Range	Participants						
					#1	#2	#3	#4	#5	#6	
Registration	33.70 (0.52)	33‐34	203.00 (61.96)	135‐285	Pass	Pass	Pass	Pass	Pass	Pass	100
Appointment booking	4.00 (0.00)	4‐4	30.20 (3.76)	27‐37	Pass	Pass	Pass	Pass	Pass	Pass	100
Telemedicine consultation	21.20 (1.94)	20‐25	204.80 (69.44)	107‐294	Pass	Pass	Pass	Pass	Pass	Pass	100
Address entry	5.80 (1.33)	5‐8	14.80 (6.82)	9‐26	Pass	Pass	Pass	Pass	Pass	Pass	100
Service history	3.00 (0.00)	3‐3	17.00 (10.18)	7‐30	Pass	Pass	Pass	Pass	Pass	Pass	100
Password reset	6.70 (0.82)	6‐8	115.20 (36.89)	72‐155	Pass	Pass	Pass	Pass	Pass	Pass	100

For the patient-facing telemedicine web application, all patient participants successfully completed the full complement of six tasks. Variability was observed in the number of interactions and task completion times, which may reflect individual differences in participants’ technological proficiency.

For the provider-facing telemedicine management platform, task success rates were similarly high, with 100% completion for most roles. The exception was the pharmacist role, which achieved a task success rate of 83.33%. The number of interactions and task completion times was not recorded for providers as the redesigned system allowed most tasks to be completed within a very short time. Variability in the pharmacist results may be attributed to individual differences in interface familiarity or task complexity (Table 3). Overall, the findings demonstrate that the provider platform was highly usable for most staff roles.

User satisfaction data are shown in Table 4. Among patients, the NPS was 67 based on feedback from 6 participants: 4 (67%) promoters, 2 (33%) passives, and no detractors. The mean satisfaction score was 9.2/10, indicating excellent user experience. For service providers, usability was evaluated using the SUS. The mean SUS score was 71.4 (SD 20.76) based on feedback from 7 participants, placing the system in the above-average usability range [34].

**Table 3.** Service provider statistical usability testing results (N=7).

Roles and tasks		Participants for each role		Success rate (%)
		1	2	
Coordinators (n=1)				100
	Task 1	Pass	—^a^	—
	Task 2	Pass	—	—
	Task 3	Pass	—	—
	Task 4	Pass	—	—
Physicians (n=2)				100
	Task 1	Pass	Pass	—
	Task 2	Pass	Pass	—
Pharmacists (n=2)				83.33
	Task 1	Fail	Pass	—
	Task 2	Pass	Pass	—
	Task 3	Pass	Pass	—
Financial personnel (n=2)				100
	Task 1	Pass	Pass	—
	Task 2	Pass	Pass	—

a Not applicable.

**Table 4.** Satisfaction scores of patients and System Usability Scale (SUS) results for service providers.

Item		Score, mean (SD)	Score, median (IQR)	Score range
Patients (n=6)				
	Knew the procedure well	8.7 (1.51)	8 (7.25‐10)	7‐10
	Completing the service felt easy	8.8 (0.98)	8 (8‐9.75)	8‐10
	Overall satisfaction with the service	9.2 (0.75)	9 (9‐9.75)	8‐10
Service providers (n=7)				
	SUS	71.4 (20.76)	65 (55‐86.25)	52.5‐100

## Discussion

### Principal Findings

This study developed and validated a new telemedicine service system for a TCM clinic centered on separate patient- and provider-facing web applications. The redesign was guided by service design tools, customer journey mapping, and service blueprinting that revealed inefficiencies in the legacy system, including fragmented communication channels, redundant steps, and undefined waiting times.

By reconfiguring these workflows into a logically harmonized digital design, the new system integrated patient-facing processes with provider operations in a coherent manner. Automation eliminated repetitive administrative tasks, communication was consolidated, and service notifications connected actions across roles. These features not only simplified the patient journey but also aligned provider tasks with patient needs in real time, reflecting a deliberate harmonization of activities across the digital service process.

Usability testing with 13 participants (n=6, 46% patients and n=7, 54% providers) demonstrated the effectiveness of this design. Patients achieved a 100% task success rate, with completion times within acceptable ranges, supporting learnability and intuitive system use [35,36]. Providers achieved similarly high success rates, with the exception of pharmacists (83.33%), whose tasks were inherently more complex. The observed errors were categorized as slips [37,38], suggesting that the design was fundamentally sound but required refinement in more information-intensive workflows, such as those for pharmacy.

The logically harmonized design also translated into high user satisfaction. Patients reported an average satisfaction score of 9.2/10 and an NPS of 67, both indicating excellent performance and strong acceptability [30,39,40]. This suggests that patients not only found the system easy to use but were also motivated to recommend it, an important marker of adoption in digital health.

Among providers, the average SUS score was 71.4 (SD 20.76), above the average usability benchmark [34]. The variability in provider scores reflected differing levels of familiarity and adaptation to the new workflows. Importantly, this underscores a central challenge of digital transformation: harmonizing not only technical systems but also user practices and mindsets. With training and support tools (eg, video tutorials, workflow guides, and quick-reference materials) [35,36], providers can adapt to the harmonized processes more readily, leading to even higher usability and acceptance.

A distinctive contribution of this study is the introduction of service notifications as an orchestration mechanism in the harmonized design. Patients received automated alerts about next steps (eg, physician availability, payment confirmation, and delivery status), whereas providers were notified when specific actions were required (eg, reviewing documents, issuing receipts, and preparing medication). These notifications bridged the frontstage and backstage operations, ensuring that both patients and providers had a shared, transparent view of the service process. This alignment reduced uncertainty, supported timely action, and reinforced the logically harmonized structure of the digital service.

Patients highlighted improvements in convenience and accessibility, particularly for those with travel barriers, whereas providers reported efficiency gains from reduced redundant tasks and better cross-role coordination. Together, these results illustrate that harmonization of digital workflows not only improves efficiency but also strengthens the interconnectedness of patient and provider experiences in telemedicine.

This study contributes to the broader discourse on digital transformation in health care by demonstrating that success lies not only in digitizing existing processes but also in logically harmonizing them across all stakeholders. The integration of customer journey maps and service blueprints provided a structured pathway to identify misalignments and redesign them into a unified digital flow.

While service design tools remain underused in TCM and telemedicine contexts [41,42], our findings show that they can be instrumental in achieving harmonized digital transformation. The resulting framework could serve as a TCM telemedicine service design model or as guidelines for telehealth service design more broadly. By bridging service design thinking with digital health practice, this study highlights how logically harmonized design enables patient-centered care, reduces provider burden, and strengthens overall health care efficiency.

### Limitations

This study faced several limitations. First, the case study clinic’s IT infrastructure constrained the technical development of the web application. For example, while Node.js supported the system backbone, the integration of Web Real-Time Communication was limited due to the complexity of configuring Traversal Using Relays Around Network Address Translators and Session Traversal Utilities for Network Address Translators servers. These challenges affected the implementation of smooth real-time communication features.

Second, the patient sample (n=6) included participants aged 23 to 54 years, which does not fully represent the broader adult population typically served by the clinic, particularly older adults (with those aged 41-50 years being the most common age group in clinic data). As older adults are likely to benefit most from remote care solutions, future telemedicine research should prioritize this demographic, focusing on usability and accessibility tailored to older users.

### Future Work

One of the distinctive challenges in TCM telehealth is the reliance on visual tongue examination for diagnosis. The current system addressed this by enabling patients to upload tongue photographs for physicians to review, as well as request resubmission if necessary. Future research could extend this by integrating machine learning and artificial intelligence to automate initial screening of tongue images. Automated feedback on image quality could reduce wasted time, decrease physician workload, and further improve diagnostic efficiency.

Beyond the TCM context, the system could be adapted for other traditional medical practices, such as Ayurveda. Although attempts to implement telemedicine in Ayurveda have been reported, barriers remain due to limited physician awareness and acceptability [43]. Applying the service design approach demonstrated in this study may support the integration of such services, particularly where workflows require specialized or customized patient data.

Finally, the use of service design tools (customer journey maps and service blueprints) in this study provides a replicable methodology for integrating new digital technologies into health care services. These tools can guide the redesign of service processes to optimize delivery, improve user experiences, and enhance both patient satisfaction and provider efficiency.

### Conclusions

This study implemented strategies for both patients and service providers to improve the telemedicine service system and streamline its operations, with a focus on reducing human effort, interaction costs, and waiting times.

For patients, 3 strategies were central: consolidating service channels into a single platform, digitizing processes through web applications, and visualizing service status. These interventions lowered interaction costs by providing an all-in-one, easily accessible platform. Visualization of service status reduced dissatisfaction from undefined waiting times and improved patient understanding of service steps. Usability testing confirmed the positive effects of these improvements, with patients reporting an NPS of 67 and an average satisfaction score of 9.2, reflecting excellent performance.

For service providers, 2 strategies were central: digitizing tasks and visualizing service status. This reduces repetitive administrative work, facilitating tracking of patient responses and supporting more efficient communication across roles. While the system markedly reduced provider workload, structured training at the start of implementation is recommended to support adoption, build confidence, and promote trust in the system.

Collectively, the results demonstrate that a logically harmonized digital transformation informed by service design methodologies can enhance telemedicine effectiveness and efficiency. By aligning redesigned workflows with patient and provider needs, the system improved usability and satisfaction while contributing to higher-quality health care delivery.

## Supplementary material

10.2196/76752 Multimedia Appendix 1 Enlarged image of the “to be” service blueprint of the redesigned telemedicine service (provider perspective).

10.2196/76752 Multimedia Appendix 2 Enlarged image of the patient web application interface optimized for mobile devices.

## Abbreviations

NPS: net promoter score
SUS: System Usability Scale
TCM: traditional Chinese medicine

## References

[1] Shirzadfar H, Lotfi F (2017). The evolution and transformation of telemedicine. Int J Biosens Bioelectron.

[2] Telemedicine: opportunities and developments in member states: report on the second global survey on ehealth. World Health Organization.

[3] Ftouni R, AlJardali B, Hamdanieh M, Ftouni L, Salem N (2022). Challenges of telemedicine during the COVID-19 pandemic: a systematic review. BMC Med Inform Decis Mak.

[4] Strehle EM, Shabde N (2006). One hundred years of telemedicine: does this new technology have a place in paediatrics?. Arch Dis Child.

[5] Loh PK, Donaldson M, Flicker L, Maher S, Goldswain P (2007). Development of a telemedicine protocol for the diagnosis of Alzheimer’s disease. J Telemed Telecare.

[6] Mishra V, Sharma MG (2022). Telemedicine as frugal intervention to health care: a case of diabetes management. Int J Healthc Manag.

[7] Watson S (2020). Telehealth: the advantages and disadvantages. Harvard Health Publishing.

[8] Smith WR, Atala AJ, Terlecki RP, Kelly EE, Matthews CA (2020). Implementation guide for rapid integration of an outpatient telemedicine program during the COVID-19 pandemic. J Am Coll Surg.

[9] Kapoor S, Eldib A, Hiasat J (2020). Developing a pediatric ophthalmology telemedicine program in the COVID-19 crisis. J AAPOS.

[10] Anthony Jnr. B (2021). Integrating telemedicine to support digital health care for the management of COVID-19 pandemic. Int J Healthc Manag.

[11] Koonin LM, Hoots B, Tsang CA (2020). Trends in the use of telehealth during the emergence of the COVID-19 pandemic - United States, January-March 2020. MMWR Morb Mortal Wkly Rep.

[12] Seivert S, Badowski ME (2021). The rise of telemedicine: lessons from a global pandemic. EMJ Innov.

[13] Ma Q, Sun D, Tan Z (2022). Usage and perceptions of telemedicine among health care professionals in China. Int J Med Inform.

[14] Sajid M, Zakkariya KA, Peethambaran M (2023). Predicting virtual care continuance intention in the post-COVID world: empirical evidence from an emerging economy. Int J Healthc Manag.

[15] Rosenbaum MS, Otalora ML, Ramírez GC (2017). How to create a realistic customer journey map. Bus Horiz.

[16] Kalbach J (2016). Mapping Experiences.

[17] Bitner MJ, Ostrom AL, Morgan FN (2008). Service blueprinting: a practical technique for service innovation. Calif Manage Rev.

[18] Chokshi SK, Mann DM (2018). Innovating from within: a process model for user-centered digital development in academic medical centers. JMIR Hum Factors.

[19] Ingaldi M, Klimecka-Tatar D (2022). Digitization of the service provision process - requirements and readiness of the small and medium-sized enterprise sector. Procedia Comput Sci.

[20] Vial G (2019). Understanding digital transformation: a review and a research agenda. J Strateg Inf Syst.

[21] Chu H, Westbrook RA, Njue-Marendes S, Giordano TP, Dang BN (2019). The psychology of the wait time experience - what clinics can do to manage the waiting experience for patients: a longitudinal, qualitative study. BMC Health Serv Res.

[22] Lallemand C, Gronier G Enhancing user experience during waiting time in HCI: contributions of cognitive psychology.

[23] Figma design. Figma.

[24] Chen PPS (1976). The entity-relationship model—toward a unified view of data. ACM Trans Database Syst.

[25] Jiamsanguanwong A, Ophaswongse C, Chansirinthorn C, Kitirattragarn N, Kittithreerapronchai O (2023). Improving patients’ experience concerning insufficient informational flow to patients during COVID-19 pandemic: case study of a traditional Chinese medicine clinic. Int J Healthc Manag.

[26] Jiamsanguanwong A, Tientrakul P, Sookseng F, Ophaswongse C, Kittithreerapronchai O (2021). System improvement of medicine delivery service: case study of traditional Chinese medicine. Int J Healthc Manag.

[27] Etikan I, Musa SA, Alkassim RS (2016). Comparison of convenience sampling and purposive sampling. Am J Theor Appl Stat.

[28] Rovinelli RJ, Hambleton RK (1977). On the use of content specialists in the assessment of criterion-referenced test item validity. Tijdschr Onderwijs Res.

[29] Frøkjær E, Hertzum M, Hornbæk K Measuring usability: are effectiveness, efficiency, and satisfaction really correlated?.

[30] Reichheld FF (2003). The one number you need to grow. Harv Bus Rev.

[31] Brooke J, Jordan PW, Thomas B, Weerdmeester B, McClelland AL (1996). Usability Evaluation in Industry.

[32] (2001). Common industry format for usability test reports, ANSI-NCITS 354–2001. National Institute of Standards and Technology.

[33] Eysenbach G, CONSORT-EHEALTH Group (2011). CONSORT-EHEALTH: improving and standardizing evaluation reports of Web-based and mobile health interventions. J Med Internet Res.

[34] Hyzy M, Bond R, Mulvenna M (2022). System usability scale benchmarking for digital health apps: meta-analysis. JMIR Mhealth Uhealth.

[35] Nielsen J (1993). Usability Engineering.

[36] Nielsen J Enhancing the explanatory power of usability heuristics.

[37] Lewis C, Norman D, Norman DA, Draper SW (1986). User Centered System Design: New Perspectives on Human–Computer Interaction.

[38] PRüMPER J, Zapf D, Brodbeck FC, Frese M (1992). Some surprising differences between novice and expert errors in computerized office work. Behav Inf Technol.

[39] Bevan N, Kirakowski J, Maissel J Human aspects in computing, design and use of interactive systems and work with terminals. https://www.hci.international/index.php?module=conference&CF_op=view&CF_id=17.

[40] (2022). Australian Healthcare Index report November 2022. Australian Healthcare Index.

[41] Joseph AL, Kushniruk AW, Borycki EM (2020). Patient journey mapping: current practices, challenges and future opportunities in healthcare. Knowl Manag e-Learn.

[42] Joseph AL, Monkman H, Kushniruk A, Quintana Y (2023). Exploring patient journey mapping and the learning health system: scoping review. JMIR Hum Factors.

[43] Azeez K K NA, Krishnan A, H K S (2023). Telemedicine usage among ayurvedic physicians: current scenario - a cross-sectional study. Int J Exp Res Rev.

