# Supplementary figures and images for: Redesigning Telemedicine for Traditional Chinese Medicine: Service Design Approach to Digital Transformation
Source: JMIR Hum Factors. 2025 Oct 28;12:e76752. doi: 10.2196/76752 (PMC12560961; doi:10.2196/76752)

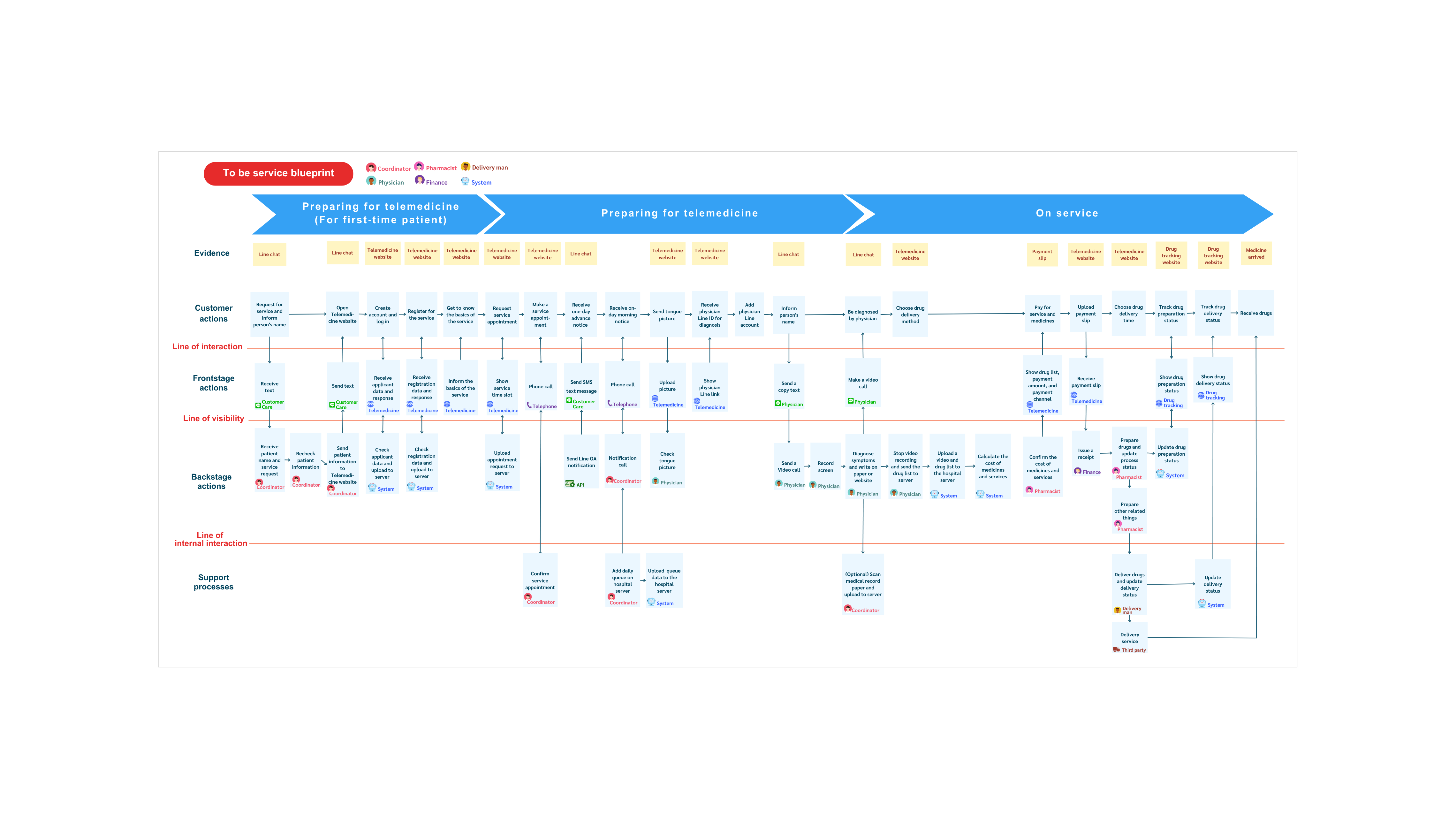

Supplement: Multimedia Appendix 1 [file humanfactors-v12-e76752-s001.png]

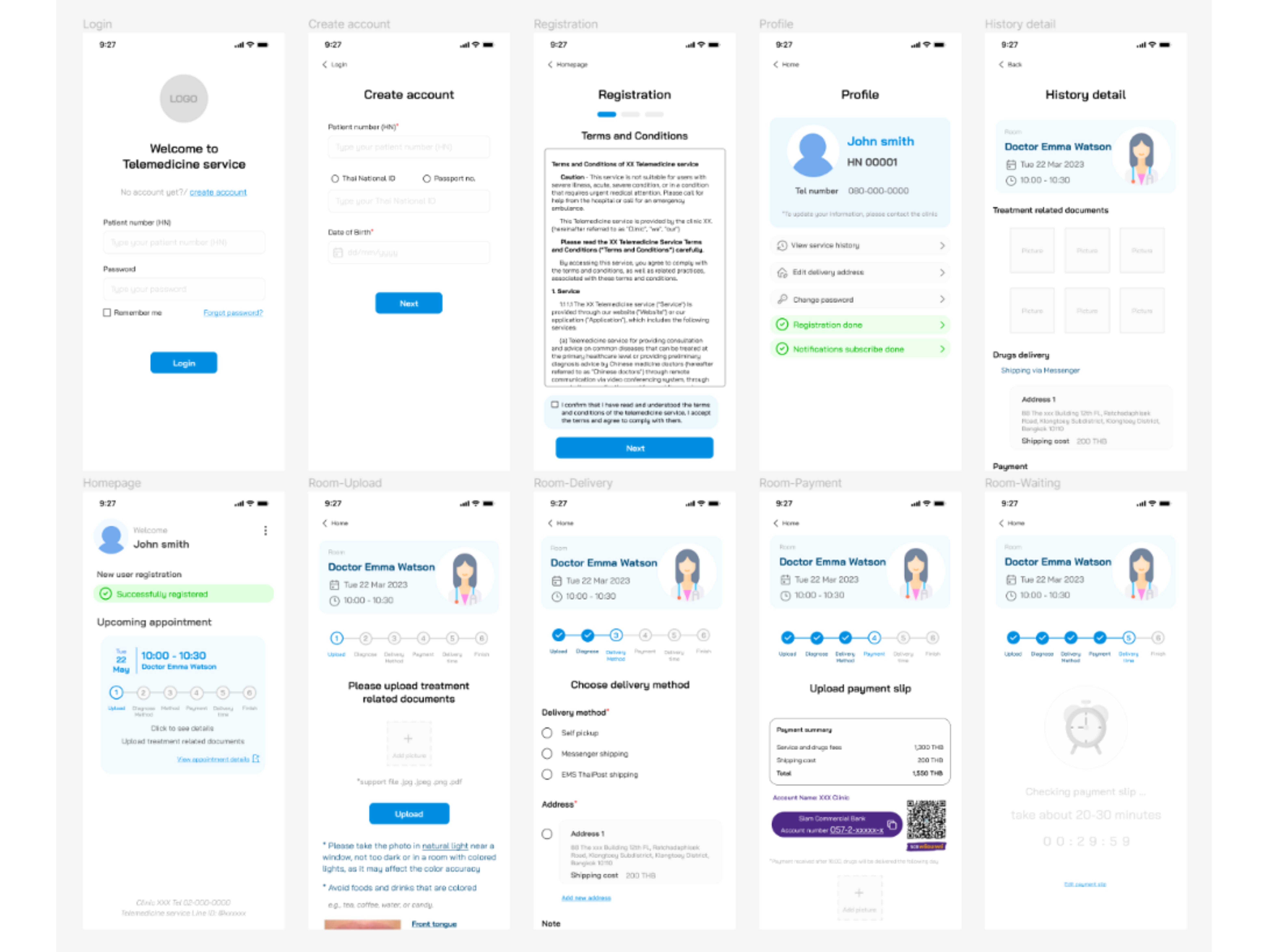

Supplement: Multimedia Appendix 2 [file humanfactors-v12-e76752-s002.png]
